# Advanced Technologies for Processing Functional Biomaterials

**DOI:** 10.3390/jfb17020091

**Published:** 2026-02-13

**Authors:** Daniel Sola

**Affiliations:** 1 Aragonese Foundation for Research and Development (ARAID), 50018 Zaragoza, Spain; dsola@unizar.es; 2 Instituto de Nanociencia y Materiales de Aragón, Universidad de Zaragoza-CSIC, 50018 Zaragoza, Spain

Biomaterial processing is a crucial operation that involves mechanical and chemical treatments to transform a source material into a biocompatible and bioactive product tailored to a specific medical application. In practice, it enables controlled changes in bulk and surface properties, the definition of targeted shapes/architectures, and improved biological performance. Given that biomaterials encompass metals, polymers, ceramics, and hybrids, their the methodologies for processing them are naturally varied. Metallic biomaterials typically undergo alloying and thermomechanical treatments, while polymers are frequently processed via injection molding, melt extrusion, and electrospinning. Additionally, porous architectures are commonly achieved through foaming or particle-leaching techniques. The fabrication of 3D structures is particularly important in tissue engineering, where approaches such as freeze drying, gas foaming, electrospinning, additive manufacturing, and laser-assisted techniques are frequently combined to engineer multiscale porous architectures and interfaces [1].

In this context, advanced processing is increasingly viewed as the central design pillar connecting composition to functionality. This approach controls multiscale architectures, including porosity, interconnectivity, and fiber organization, as well as interfacial cues such as topography and wettability, which ultimately determine mechanical and biological performance. While additive manufacturing and biofabrication represent new methods of biomaterial processing, their success relies on precisely managing printability and shape retention. These factors are closely governed by the chemical formulation and the rheology of the ink or bio-ink [2]. In parallel, ultrashort-pulse laser surface structuring, particularly on substrates used for medical implants such as zirconia, offers a non-contact method for generating hierarchical micro/nanotextures with demonstrated potential to tune cell response and antibacterial performance, depending on texture regimes and processing conditions [3].

This Special Issue brings together eight contributions that can be grouped into four complementary themes. First, we present a group of papers focused on biofabrication and ECM-mimicking 3D microenvironments, including spheroid–exosome bioprinting concepts and electrohydrodynamic methods of fabricating nanofibrous culture substrates and scaffolds. Second, we highlight additive manufacturing and processability–performance relationships, including printability/rheology in biodegradable polymer blends and the biological evaluation of additively manufactured bioceramic scaffolds. Third, the engineering of surface interfaces in medical implants is represented through green electrospun antibacterial coatings and femtosecond-laser texturing strategies that tune surface morphology and topography. Finally, a transversal contribution illustrates processing-enabled multifunctionality, where directional solidification in a bioactive coating induces microstructural anisotropy and a polarization-dependent optical response.

The first thematic cluster of this Special Issue focuses on biofabrication strategies and ECM-mimicking microenvironments aimed at improving the physiological relevance of in vitro models and enabling more instructive 3D culture platforms. Across these contributions, processing is treated as a tool to control not only geometry and nanoarchitecture, but also the biological signals that govern cell adhesion, organization, and long-term behavior.

Lee and Lee review the emerging convergence between bioprinting, spheroid assembly, and exosome biology, highlighting spheroids as a scalable 3D cell culture unit suitable for mass production through bioprinting and for sustained exosome secretion. The authors provide evidence that spheroid-based constructs facilitate volumetric tissue reconstruction by utilizing exosomes as paracrine mediators to drive regeneration, and they discuss the practical bottlenecks for translation, including spheroid culture control, exosome isolation and purification, and long-term storage and dosing strategies [4].

Tran et al. [5] address a key limitation of highly hydrophilic electrospun PVA matrices, namely, the loss of fibrous morphology upon exposure to water, by introducing chemical crosslinking and post-crosslinking to obtain a water-stable, porous, and optically transparent PVA nanofibrous membrane for 3D culture. Recognizing that synthetic PVA lacks intrinsic cell-binding motifs, the study further incorporates integrin-binding peptides such as fibronectin- and laminin-derived sequences, retained within the matrix to promote peptide-specific adhesion and growth patterns closer to those exhibited by the ECM. Crucially, these peptide-functionalized membranes do not provoke a response from dendritic cells, confirming their potential as bioinert substrates for ECM replacement.

Complementing electrospinning-based membranes, Ikeuchi et al. [6] propose a scalable polymer-processing method that integrates nanofibrous surface formation with mold-defined shaping: phase-separation-assisted electrospray of PLA generates microcapsules with ECM-like porous nanofibrous surfaces, which are then deposited onto conductive molds to reproduce 2D/3D geometries with reported feature resolution as low as ~200 µm and scaffold thickness up to ~600 µm. The resulting scaffolds support strong HepG2 adhesion and metabolic activity. However, cell distribution remains largely confined near the surface (with penetration limited to ~80 µm), underscoring a persistent challenge for thick constructs, transport, and infiltration, and indicating the need for future work on pore connectivity, microcapsule packing, and dynamic culture strategies.

The second thematic cluster highlights additive manufacturing as a method for generating controlled, application-driven geometries, while emphasizing that reliable performance depends on understanding processability constraints and their translation to microstructure and biological response. The two papers in this block connect polymer melt morphology and rheology to printability and dimensional fidelity, and also connect the manufacturing of bioceramic scaffolds to multi-assay biological evaluation.

Costantini et al. [7] examine the interplay among melt morphology, rheology, and fused deposition modeling (FDM) printability in poly(lactic acid)/poly(3-hydroxybutyrate-co-3-hydroxyvalerate) (PLA/PHBV) blends. By correlating blend composition with viscoelastic properties, extrusion behavior, and print outcomes, the study provides a process–property framework to rationally define printing windows and improve shape fidelity for biodegradable thermoplastic feedstocks. Importantly, the study illustrates how rheological descriptors can be used to anticipate filament stability and geometric accuracy, supporting more reproducible fabrication of complex architectures.

Shan et al. [8] report in vitro biological characterization of 3D-printed and sintered hydroxyapatite scaffolds obtained by fused filament fabrication. Using complementary readouts such as cell morphology and distribution, viability/proliferation and cytotoxicity, osteogenic gene expression, and protein synthesis, the authors demonstrate overall cytocompatibility and osteogenic differentiation potential while also highlighting test-related constraints, such as transient cytotoxic signals, which may arise under static culture conditions. The study therefore illustrates both the promise of additively manufactured bioceramic architectures and the importance of robust, physiologically relevant evaluation protocols for translation.

The third thematic cluster focuses on the engineering of interfaces in medical implants, where surface chemistry and micro/nanotopography critically influence tissue integration and bacterial colonization. The two contributions in this group represent complementary processing methods: electrospinning-based coatings that embed antibacterial function, and femtosecond-laser texturing that tunes the topography on clinically relevant substrates without altering bulk properties.

Caporalini et al. [9] review electrospinning as a versatile technique for applying antibacterial coatings to medical devices, emphasizing how high surface area, tunable porosity, and controllable incorporation/release of antimicrobial agents can be leveraged to mitigate infection risk. A particular focus is placed on “green electrospinning” strategies, including bio-based polymers and less-toxic solvent systems, as well as natural antimicrobial compounds, framing sustainability as an increasingly relevant design constraint alongside efficacy. The review also discusses practical barriers for translation, such as coating durability, scalable manufacturing, and standardized antibacterial testing protocols.

Hartung et al. [10] investigate femtosecond-laser processing of glass solder-coated zirconia substrates, and demonstrate that surface morphology and topography can be steered across distinct regimes by adjusting pulse energy and scanning/overlap parameters. The study reports reproducible formation of wave-like textures at lower pulse energy and cauliflower-like morphologies at higher energy, highlighting the role of pulse-to-pulse and line-to-line overlap in determining microstructural features. These results support the use of the proposed method as a parameter-driven route to tailoring zirconia-based implant interfaces, with potential implications for tissue ingrowth and reduced bacterial adhesion.

A transversal contribution in this Special Issue highlights a broader direction for research in this field: embedding additional functionalities such as optical readouts into biomaterials through processing-induced microstructural control. Such approaches may enable contactless tracking or monitoring concepts while preserving core bioactive performance.

Sola et al. [11] conduct a study on neodymium-doped bioactive wollastonite–tricalcium phosphate (W–TCP:Nd) coatings produced via dip-coating followed by laser-floating-zone directional solidification. Micro-Raman spectroscopy demonstrate processing-induced anisotropy between longitudinal and transversal sections, while polarization-resolved photoluminescence shows that the ~1060 nm transition is nearly polarization-independent, whereas the 870–900 nm band exhibits Stark splitting and pronounced polarization dependence. The study therefore demonstrates how directional solidification can imprint optical anisotropy into a bioactive coating, indicating its potential for polarization-based optical tracking or sensing concepts in functional implant coatings.

The contributions in this Special Issue collectively reinforce the idea that processing is becoming the primary design axis that connects composition to functional performance in biomaterials. Across biofabrication, electrohydrodynamic scaffold formation, additive manufacturing, and laser surface engineering, these papers illustrate how controlling architectures and interfaces, often across multiple length scales, can directly shape biological outcomes (cell adhesion, differentiation, tissue integration) and device-relevant functions (antibacterial behavior or optical readouts). Importantly, several works also highlight the practical bottlenecks that limit translation, such as printability constraints, thickness-dependent mass transport in 3D constructs, and the sensitivity of biological conclusions to testing conditions.

Looking forward, research in this field will benefit from standardized, cross-platform metrics that link processing parameters to structure (pore interconnectivity, infiltration depth, surface feature descriptors) and to function (biological response and anti-biofilm performance) in a reproducible way. Equally important will be the adoption of more physiologically relevant validation workflows, including dynamic culture/perfusion for thick scaffolds, long-term stability and durability assessments for coatings, and harmonized antibacterial/biofilm models. Finally, multifunctional concepts such as polarization-dependent optical responses present an emerging opportunity to embed non-contact monitoring capabilities into bioactive materials, enabling smarter biomaterial platforms where integrity-, orientation-, or degradation-related changes can be tracked alongside therapeutic performance.
